# Stereotactic ablative body radiotherapy boost for cervical cancer when brachytherapy boost is not feasible

**DOI:** 10.1186/s13014-021-01877-4

**Published:** 2021-08-12

**Authors:** Tae Hoon Lee, Changhoon Song, In Ah Kim, Jae-Sung Kim, Yong Beom Kim, Kidong Kim, Jae Hong No, Dong Hoon Suh, Jin-Beom Chung, Keun-Yong Eom

**Affiliations:** 1 Department of Radiation Oncology, Seoul National University Hospital, Seoul, Republic of Korea; 2 Department of Radiation Oncology, Seoul National University Bundang Hospital, 82 Gumi-ro 173 beon-gil, Bundang-gu, Seongnam, 13620 Republic of Korea; 3 Department of Obstetrics and Gynecology, Seoul National University Bundang Hospital, Seongnam, Republic of Korea

**Keywords:** Cervical cancer, Stereotactic body radiotherapy, Hematuria, Hematochezia

## Abstract

**Background:**

The purpose of this study was to analyze the treatment efficacy and safety of stereotactic ablative body radiotherapy (SABR) boost for cervical cancer patients not amenable to brachytherapy.

**Methods:**

A retrospective review of the medical records from single institution of 25 eligible patients was performed. The patients underwent pelvic radiotherapy (RT) in 25 or 28 fractions with a median dose of 45 Gy (range 44–50.4 Gy). SABR boost was delivered after pelvic RT, with a median dose of 25 Gy (range 20–33 Gy), and a median fraction number of 5 (range 4–6). 21 patients with a follow-up period of more than one year were included in the toxicity analysis, and hematuria and hematochezia that occurred later than 3 months after the RT were graded.

**Results:**

The median follow-up period after radiotherapy was 2.85 years (range 0.33–6.60). The 3-year local control, locoregional control, disease-free survival, and overall survival rates were 80.9%, 75.8%, 40.9%, and 77.1%, respectively. 5 patients experienced grade 3 toxicity (3 genitourinary, 3 gastrointestinal), and no grade 4–5 toxicity was reported. Univariate analysis showed that cumulative D_2cc_ in equivalent dose in 2 Gy fractions (EQD2) of rectum was marginally predictive for any grade of hematochezia (*P* = 0.051). Cumulative D_2cc_ EQD2 of bladder was not predictive for hematuria. In the receiver operating characteristic (ROC) curve analysis, the optimal threshold of cumulative rectal D_2cc_ EQD2 was 81.2 Gy for any grade of hematochezia.

**Conclusion:**

SABR boost for cervical cancer was effective and tolerable. Although it cannot substitute brachytherapy, it can be a treatment option when brachytherapy is not possible.

## Introduction

Patients with locally advanced cervical cancer are usually treated with concurrent chemoradiation and brachytherapy boost. It is well known that brachytherapy is an essential component of treatment for locally advanced cervical cancer, and some reports have shown that brachytherapy is associated with better treatment outcomes, including cancer-specific survival [1]. Despite the importance of brachytherapy, the availability of brachytherapy is relatively limited because it is operator dependent, and failure to maintain the quality of brachytherapy can reduce the treatment efficacy [2]. In addition, a small proportion of patients cannot undergo brachytherapy due to an obstructing tumor mass, anatomical variations, or medical comorbidities. For patients who are unable to undergo brachytherapy boost due to the aforementioned reasons, external beam radiotherapy (EBRT) boost can be an alternative. Some studies with small sample sizes evaluated EBRT boost, and reported that the outcomes were sufficiently acceptable to consider EBRT boost when brachytherapy is not available [3, 4].

Recently, stereotactic ablative body radiotherapy (SABR) has been applied for the treatment of several tumors arising from various organs. Compared with cervical EBRT boost reported in earlier studies, a higher biologically equivalent dose can be delivered by the SABR technique. Additionally, as SABR can deliver a higher dose per fraction, the proposed dose-fractionation schemes for cervical SABR boost are more similar to the schemes for brachytherapy when comparing with those for EBRT boost with conventional fractionation [5], although central dose would still be limited. As a result, cervical SABR boost is a promising option for cervical cancer which is not amenable to brachytherapy boost; nevertheless, the feasibility and safety of SABR boost should be evaluated. The purpose of this study was to analyze the treatment efficacy and toxicity of SABR boost in cervical cancer patients.

## Methods

### Study population

The medical records of patients from single institution with histologically confirmed cervical cancer who underwent curative radiotherapy (RT) with SABR boost between 2013 and 2019 were retrospectively reviewed, and 25 patients were found to be eligible. These patients were not suitable for brachytherapy boost and had no prior pelvic RT. Among them, 20 (80.0%) patients were unable to undergo brachytherapy due to failure of intracavitary device insertion because of a narrow vagina or cervical os fibrosis. These patients found to be ineligible to undergo brachytherapy after cervical os cannulation effort by an experienced gynecologist. Two (8.0%) patients had large tumors that could not be adequately irradiated by brachytherapy. One patient had extreme obesity, making the evaluation of the cervix and uterine canal impossible. Among the remaining two patients, one patient experienced intolerable pain with an intracavitary device, while the other patient was unable to position appropriately due to left spastic hemiplegia.

### Pelvic radiotherapy and chemotherapy

All patients underwent whole pelvic RT. For all but one patient, the radiation dose to the pelvis was 45–50.4 Gy, and the dose per fraction was 1.8 Gy. One patient had a pelvic dose of 44 Gy, and the dose per fraction was 2 Gy. Both 3-dimensional conformal radiation therapy (3D-CRT) and intensity-modulated radiation therapy (IMRT) techniques were used, but IMRT was applied to only two (8.0%) patients. For all cases, the clinical target volume (CTV) was delineated based on previously published consensus guidelines [6]. As stated in the guidelines, the cervical CTV included all gross tumors, cervix, uterus, parametria, and upper vagina, while the nodal CTV included the common iliac, internal iliac, external iliac, obturator, and presacral lymph nodes. If suspected metastatic lymph nodes in the common iliac lymph node area or above were detected by staging examinations, para-aortic lymph nodes were added to the nodal CTV. The pelvic planning target volume (PTV) was calculated by adding a 5 mm margin around the cervical and nodal CTV. For the 3D-CRT technique, “box field” consisting of parallel opposed AP-PA fields and two lateral opposed fields with 15 MV photon beams was set up to cover the pelvic PTV. For IMRT, the volumetric-modulated arc therapy (VMAT) technique was applied, and two 360-degree arcs of beams with a 10 MV photon beam were used. Boost to gross lymph nodes was added in patients with suspected metastatic lymph nodes. The lymph node boost was delivered sequentially in eight (32.0%) patients, and concurrently in two (8.0%) patients using IMRT plans by a simultaneous integrated boost technique. The radiation dose for the lymph node boost was variable (range 5.4–14.4 Gy). For consistent bladder filling, the patients were instructed to restrain from voiding urine for one hour after the last voiding and drink two cups of water.

During pelvic RT, concurrent cisplatin was administered to medically fit patients. A weekly cisplatin regimen was applied to 12 (48.0%) patients, and a tri-weekly cisplatin regimen was applied to five (20.0%) patients. For patients underwent weekly cisplatin regimen, the median number of chemotherapy cycle was 6 (range 4–7). Among these patients, 10 patients were able to complete planned chemotherapy. For patients underwent tri-weekly cisplatin regimen, the median number of chemotherapy cycle was 3 (range 2–3), and 4 patients were able to complete planned chemotherapy. Four patients underwent consolidation chemotherapy after concurrent chemoradiation. In three patients, the consolidation regimen consisted of paclitaxel and cisplatin with or without bevacizumab. One patient underwent a cycle of consolidation tri-weekly cisplatin after concurrent chemoradiation.

### SABR boost and follow-up

After completing pelvic RT, the patients underwent SABR boost to the cervix. The patients underwent a simulation computed tomography (CT) scan for SABR boost 1 week before the end of pelvic RT. To reduce spatial variability from the adjacent organs, a Foley catheter was inserted and 150–200 cc of normal saline was infused into the bladder to maintain a reproducible bladder volume, along with emptying of the rectum by an enema. These procedures were performed before the simulation CT scan and repeated for every fraction. Pelvic magnetic resonance imaging (MRI) was performed in the same day with RT simulation, and the MRI image was fused with the simulation CT scan.

**Fig. 1:**
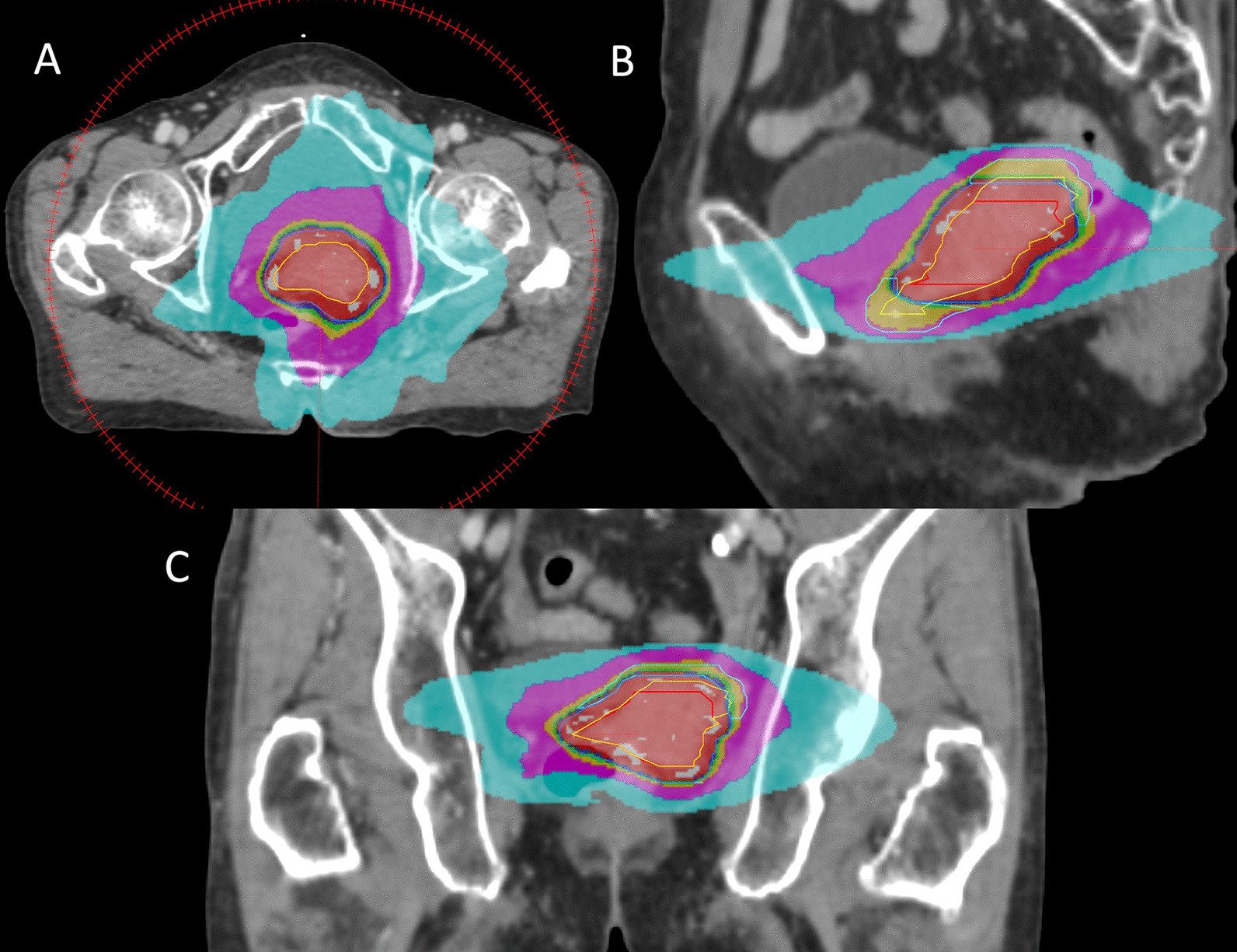
An example of cervical SABR boost plan. (**a**) Axial, (**b**) sagittal, and (**c**) coronal cut of representative SABR plan. Red, yellow, blue and cyan lines indicate CTV_25Gy_, CTV_20Gy_, PTV_25Gy_ and PTV_20Gy_, respectively

Regarding the dose-fractionation scheme and target volume delineation, an institutional protocol was implemented in late 2014. Before this, dose-fractionation was variable (range, total 20–33 Gy, 4–6 fractions), and the exact procedure to delineate the target volume was inconsistent. Fifteen patients were treated based on the institutional protocol. Dose-fractionation was 25 Gy in 5 fractions for these patients, except for two patients who received 20 Gy in 4 fractions due to unacceptable organ at risk (OAR) dose. The principles of target volume delineation in the protocol were as follows. The gross tumor volume (GTV) included residual tumor in pre-SABR image. The clinical target volume for the 25 Gy area (CTV_25Gy_) was GTV plus the entire cervix. CTV_25Gy_ was expanded by 5 mm in all directions to make the planning target volume for the 25 Gy area (PTV_25Gy_). CTV_20Gy_ was defined as CTV_25Gy_ with a 10 mm margin expansion in the superior and inferior directions. PTV_20Gy_ was constructed by 5 mm expansion of CTV_20Gy_. Rectal V25 of SABR was limited below 25%. For planning SABR, the VMAT technique with two 360-degree arcs with a 10 MV photon beam was applied, except for one patient who was treated with a static IMRT plan. An example of SABR plan was illustrated in Fig. 1.

The patients were followed up with clinical examination, tumor marker test and radiologic examination at 1 month after RT. Thereafter, patients were recommended to follow-up by clinical examination and tumor marker test with 3-month interval and radiologic examination with 3- to 6-month interval for 2 years. The follow-up interval was doubled for the third to fifth years. Tumor marker test included SCC, CA-125, and CEA if the tumor pathology was adenocarcinoma. Radiological examination included CT, MRI, or positron emission tomography (PET)/CT scan.

### Clinical outcomes and toxicity profile

The clinical outcomes in this study were local control (LC), locoregional control (LRC), progression-free survival (PFS), and overall survival (OS), which were calculated using the Kaplan–Meier method. These treatment outcomes were measured from the date of the end of RT for each defined event. An LC event was defined as a recurrence in the treated cervix or parametrium, while an LRC event was defined as a recurrence in the pelvic area. For OS, the event was defined as the death of the patient, whereas for PFS, the event was defined as disease progression or death.

For analysis of RT toxicity, medical records of the patients with a follow-up period of more than one year were reviewed, and 21 patients were included. New occurrences or worsening of the toxicities more than 3 months after the completion of RT were recorded and graded by the Common Terminology Criteria for Adverse Events (CTCAE) version 5. Most reported adverse events were genitourinary and gastrointestinal toxicities. Hematuria and hematochezia were also relatively well-documented throughout the medical records. Therefore, we classified the toxicities into five categories: urinary symptoms (other than hematuria), hematuria, proctitis (other than hematochezia), hematochezia, and others. Additionally, the rate of freedom from toxicity was calculated for hematuria and hematochezia using the Kaplan–Meier method. For dose–response analysis, cumulative dose delivered to 2 cc volume (D_2cc_) of the bladder and rectum was used. Cumulative D_2cc_ was calculated by adding equivalent dose in 2 Gy fractions (EQD2) with α/β of 3 of prescribed dose-fractionation of pelvic RT to EQD2 of D_2cc_ of bladder and rectum from the SABR plan. Univariate analysis with logistic regression was performed to find whether the cumulative D_2cc_ of rectum and bladder was predictive for occurrence of hematochezia and hematuria. Receiver operating characteristic (ROC) curves were created for the toxicities with cumulative D_2cc_ of these OARs to determine the threshold dose when the univariate analysis showed statistical significance. The optimal threshold of cumulative D_2cc_ was calculated using the Youden index method. All statistical analyses were performed using R 3.6.0 (The R Foundation for Statistical Computing, Vienna, Austria).

## Results

### Patient characteristics and radiotherapy specifics

The characteristics of all patients included in this study are summarized in Table 1. The median follow-up period for all patients was 2.85 years (range 0.33–6.60 years). The median age at diagnosis was 73.7 years, and the average was 67.7 years (range, 29.6 to 88.2 years). Most patients (80.0%) had good performance status ranging from 0 to 1 using the Eastern Cooperative Oncology Group (ECOG) performance status scale, although some (20.0%) patients with poor performance status were also included. More than half of the patients (56.0%) had cervical cancer with International Federation of Gynecology and Obstetrics (FIGO) stage III or IV. Two patients had distant metastasis at the start of RT. One patient had supraclavicular lymph node metastasis, and supraclavicular lymph node was also included in the RT field. Another patient had a questionable metastatic lesion in the liver in initial MRI. Re-evaluation of the suspected hepatic lesion after concurrent chemoradiation was planned, and the lesion progressed 3 months after the completion of concurrent chemoradiation. Additional chemotherapy was not administered due to refusal of the further treatment.

**Table 1:** Patient characteristics

Characteristics	Number
Total	25 (100.0%)
Age (years, median)	73.7 (range 29.6–88.2)
ECOG performance status	
0	1 (4.0%)
1	19 (76.0%)
2	4 (16.0%)
3	1 (4.0%)
FIGO stage (2018)	
I	1 (4.0%)
II	10 (40.0%)
III	9 (36.0%)
IV	5 (20.0%)
Pathology	
Squamous cell carcinoma	20 (80.0%)
Adenocarcnoma	3 (12.0%)
Poorly differentiated carcinoma	2 (8.0%)
Pelvic organ invasion	
Yes	4 (16.0%)
No	21 (84.0%)
Pelvic lymph node metastasis	
Yes	9 (36.0%)
No	16 (64.0%)
Paraaortic lymph node metastasis	
Yes	6 (24.0%)
No	19 (76.0%)
Distant metastasis	
Yes	2 (8.0%)
No	23 (92.0%)
Concurrent chemotherapy	
Yes	17 (68.0%)
No	8 (32.0%)
Consolidation chemotherapy	
Yes	4 (16.0%)
No	21 (84.0%)

ECOG, eastern cooperative oncology group; FIGO, International Federation of Gynecology and Obstetrics

The specifics of pelvic RT and SABR boost are summarized in Table 2. The median dose and fractionation for pelvic RT was 45 Gy in 25 fractions. For the SABR boost, the median dose and fractionation were 25 Gy and 5 fractions, respectively, according to the institutional protocol. For simple summation of biologically effective dose (BED) with α/β of 10 from the prescribed dose fractionation scheme of pelvic RT and SABR boost, the median was 90.6 Gy (range 83.1–110.6 Gy). The median EQD2 was 75.5 Gy (range 69.3–92.2 Gy), which is lower than the recommended EQD2 for adding brachytherapy after EBRT [7]. Median total treatment time was 52 days (range 35–68 days). 4 patients had total treatment time more than 8 weeks (56 days).

**Table 2:** Radiotherapy specifics

Pelvic RT technique	
3D-CRT	23 (92.0%)
IMRT	2 (8.0%)
Pelvic RT dose (Gy, median)	45 (range 44–50.4)
Pelvic RT fractionations (median)	25 (range 22–28)
Pelvic RT BED (Gy, median, α/β = 3)	72 (range 72–80.64)
Pelvic RT BED (Gy, median, α/β = 10)	53.1 (range 52.8–59.47)
Lymph node boost	
Sequential	8 (32.0%)
Simultaneous	2 (8.0%)
No	15 (60.0%)
Bladder (pelvic RT)	
Mean dose (Gy, average ± SD)	46.3 ± 3.8
Mean BED (Gy, average ± SD, α/β = 3)	73.9 ± 7.3
Maximum dose (Gy, average ± SD)	50.0 ± 3.4
Maximum BED (Gy, average ± SD, α/β = 3)	82.2 ± 6.9
Rectum (pelvic RT)	
Mean dose (Gy, average ± SD)	44.2 ± 3.9
Mean BED (Gy, average ± SD, α/β = 3)	69.4 ± 7.7
Maximum dose (Gy, average ± SD)	49.4 ± 2.5
Maximum BED (Gy, average ± SD, α/β = 3)	80.9 ± 4.6
SABR boost dose (Gy, median)	25 (range 20–33)
SABR boost fractions	
4	4 (16.0%)
5	17 (68.0%)
6	4 (16.0%)
SABR boost BED (Gy, median, α/β = 3)	66.7 (range 53.3–93.5)
SABR boost BED (Gy, median, α/β = 10)	37.5 (range 30–51.2)
Total volume of PTV (cc)	120.0 (range 70.9–412.1)
Bladder (SABR)	
Mean dose (Gy, average ± SD)	12.3 ± 4.4
Mean BED (Gy, average ± SD, α/β = 3)	23.1 ± 11.6
Maximum dose (Gy, average ± SD)	25.3 ± 3.1
Maximum BED (Gy, average ± SD, α/β = 3)	68.4 ± 11.0
D_2cc_ EQD2 (Gy, average ± SD, α/β = 3)	31.4 ± 6.8
Cumulative D_2cc_ EQD2 (Gy, average ± SD, α/β = 3	79.9 ± 6.1
Rectum (SABR)	
Mean dose (Gy, average ± SD)	11.4 ± 2.9
Mean BED (Gy, average ± SD, α/β = 3)	20.6 ± 7.5
Maximum dose (Gy, average ± SD)	24.5 ± 3.5
Maximum BED (Gy, average ± SD, α/β = 3)	65.2 ± 12.7
D_2cc_ EQD2 (Gy, average ± SD, α/β = 3)	34.8 ± 6.9
Cumulative D_2cc_ EQD2 (Gy, average ± SD, α/β = 3	76.5 ± 6.2

RT, radiotherapy; 3D-CRT, three dimensional conformal radiotherapy; IMRT, Intensity-modulated radiation therapy; BED, biological equivalent dose; SABR, stereotactic ablative body radiotherapy; SD, standard deviation; D_2cc_, dose delivered to 2 cc volume; PTV, planning target volune

### Treatment outcomes

**Fig. 2:**
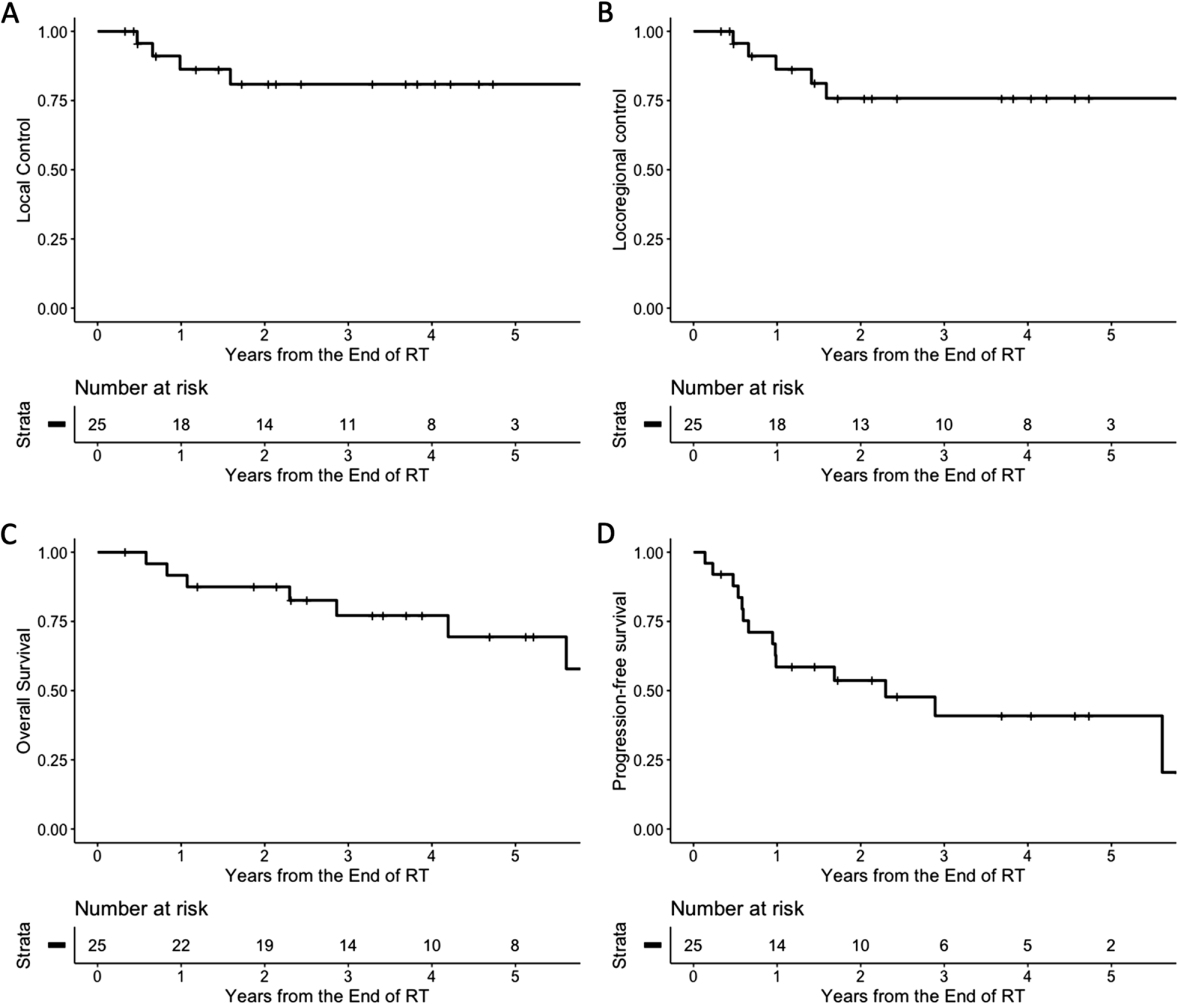
Treatment outcomes. (**a**) Local control, (**b**) locoregional control, (**c**) overall survival, and (**d**) progression-free survival of the included patients

The 1-, 3-, and 5-year LC rates were 86.3%, 80.9%, and 80.9%, respectively, and the 1-, 3-, and 5-year LRC rates were 86.3%, 75.8%, and 75.8%, respectively. The OS rates at 1, 3, and 5 years were 91.7%, 77.1% and 69.4%, respectively, while the PFS rates at 1, 3, and 5 years were 58.5%, 40.9%, and 40.9%, respectively. The most frequent failure pattern was distant failure, which occurred in eight (32.0%) patients. Four (16.0%) patients developed para-aortic lymph node metastasis after treatment. Four (16.0%) patients experienced local failure, and two (8.0%) patients had regional failure. No disease progression within the follow-up period was reported among 14 (56.0%) patients. The treatment outcomes of all patients are presented in Fig. 2.

### Toxicity profile

**Fig. 3:**
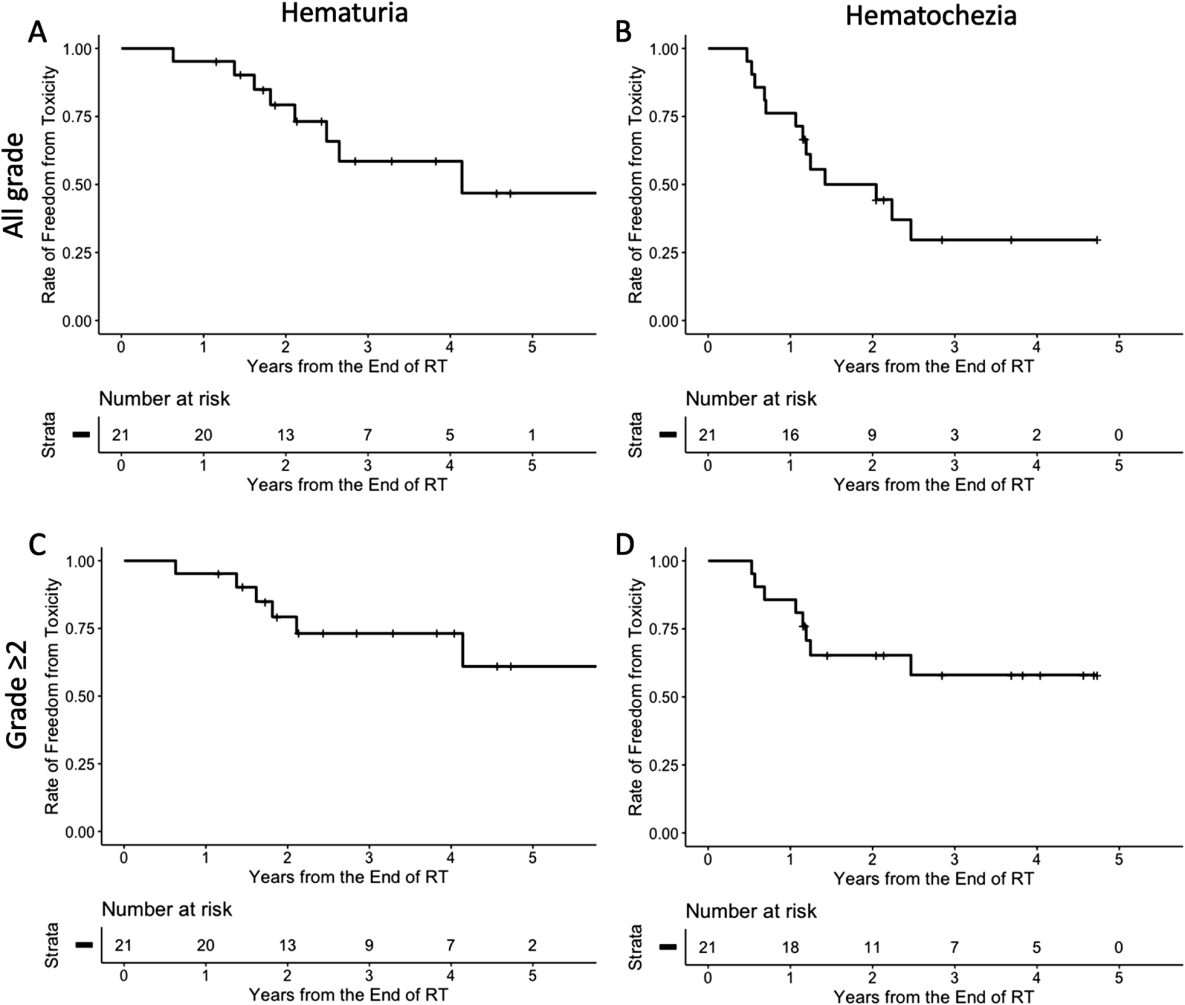
Rate of freedom from toxicity. Rate of freedom from (**a**) hematuria with any grade, (**b**) hematochezia with any grade, (**c**) hematuria with grade 2 or worse, (**d**) hematochezia with grade 2 or worse

Toxicities that occurred later than 3 months after the end of RT are summarized in Table 3. All reported toxicities were either genitourinary or gastrointestinal, with the exception of grade 2 lower extremity edema. Five (23.8%) patients had grade 3 adverse events. Three (14.3%) patients experienced grade 3 hematuria, and three (14.3%) patients experienced grade 3 hematochezia. There was no grade 4 or 5 toxicity. The median period for onset of hematuria was 23.9 months (range 7.7–50.4 months), and the median period for onset of hematochezia was 16.2 months (range 6.9–44.9 months). Among four patients with local failure, two grade 2 urinary symptoms other than hematuria, one grade 2 hematuria, one grade 1 proctitis other than hematochezia, one grade 1 hematochezia, and one grade 2 hematochezia were reported. The rates of freedom from hematuria and hematochezia are illustrated in Fig. 3. The 1-, 3-, and 5-year rates of freedom from any grade of hematuria were 95.2%, 58.5%, and 46.8%, respectively, while the 1- and 3-year rates of freedom from any grade of hematochezia were 76.2% and 29.6%, respectively. The 1-, 3-, and 5-year rates of freedom from hematuria with grade 2 or worse were 95.2%, 73.2%, and 61.0%, respectively, while the 1- and 3-year rates for hematochezia with grade 2 or worse were 85.7% and 58.0%, respectively.

**Table 3:** Toxicity profile

Grade	Type of toxicity				
	Urinary symptoms (other than hematuria)	Hematuria	Proctitis (other than hematochezia)	Hematochezia	Others
None	11 (52.4%)	13 (61.5%)	13 (61.9%)	8 (38.1%)	20 (95.2%)
1	3 (14.3%)	2 ( 9.5%)	6 (28.6%)	5 (23.8%)	0 (0.0%)
2	5 (23.8%)	3 (14.3%)	2 (9.5%)	5 (23.8%)	1 (4.8%)
3	2 (9.5%)	3 (14.3%)	0 (0.0%)	3 (14.3%)	0 (0.0%)

**Fig. 4:**
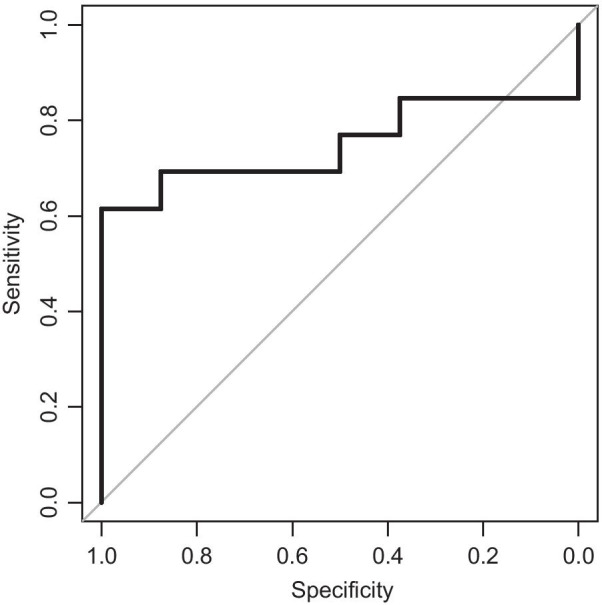
Receiver operating characteristic curve for occurrence of hematochezia. Receiver operating characteristic curve for occurrence of any grade of hematochezia by rectal cumulative D_2cc_ EQD2

In the univariate analysis, cumulative D_2cc_ of rectum was marginally correlated with occurrence of any grade of hematochezia (*P* = 0.051). No correlation was found with occurrence of grade ≥ 2 (*P* = 0.213) or ≥ 3 hematochezia (*P* = 0.127). Cumulatve D_2cc_ of bladder was not correlated with hematuria regardless of cutoff grade (any grade, *P* = 0.829; grade ≥ 2, *P* = 0.318; grade ≥ 3, *P* = 0.739). In the ROC curve analysis, cumulative D_2cc_ of rectum showed statistical significance for any grade of hematochezia (area under the curve [AUC] 0.7500, 95% confidence interval [CI] 0.5281–0.9719), and the optimal threshold was 81.2 Gy EQD2 with a sensitivity of 61.5% and specificity of 100%. The ROC curve is illustrated in Fig. 4.

## Discussion

The present study evaluated SABR boost in patients who could not undergo brachytherapy, and reported four (16.0%) local failures with a 3-year local control rate of 80.9%. The reported local failure rate was comparable to a landmark study that employed concurrent chemoradiation and brachytherapy for locally advanced cervical cancer [8], but lower than recent studies that utilized MRI-based brachytherapy [9]. Although OS and PFS rates were relatively low in the current study, it should be noted that the enrolled patients in the present study were old and morbid. For instance, more than half of the patients from GOG 120 trial had age less than or equal to 50 years, and over 90% of the patients had Karnofsky performance status more than or equal to 70 [8]. Patients from RetroEMBRACE trial had median age of 53 [9]. In the present study, median age of the patients was 73.7 years, and 20% of the patients had ECOG performance score 2–3. Toxicities were manageable in the majority of the patients, with no grade 4 or 5 adverse events.

There have been several attempts to substitute brachytherapy boost with EBRT boost even before IMRT and SABR techniques became common. Although previous EBRT boost studies utilizing the 3D-CRT technique reported acceptable treatment outcomes and toxicity profiles [10, 11], the dose delivered to the tumor was often lower than brachytherapy boost or EBRT boost with modern techniques. For instance, in a study by Barraclough et al., a total dose of 60–65 Gy with conventional fractionation was delivered to 71% of the patients [3], which is lower than the median EQD2 of 75.5 Gy in the present study. With the adoption of stereotactic techniques as well as the advancements in imaging techniques during RT, several groups have reported treatment outcomes of cervical SABR boost after pelvic RT for locally advanced cervical cancer. The reported outcomes of recent SABR boost studies are summarized in Table 4. Along with the present study, these studies revealed an acceptable local control rate of 70 to 100%, indicating that SABR boost may be sufficiently effective for cervical disease control, although a wide range of OS and PFS rates have been reported depending on the baseline patient status. It should be noted that patients who undergo cervical SABR boost tend to be older and have more comorbidities than other locally advanced cervical cancer patients who require brachytherapy boost [18].

**Table 4:** Reported outcomes of recent cervical stereotactic ablative body radiotherapy boost studies

Authors	Study type	Number of patients	Median follow-up (months)	Pelvic RT total dose (Gy)	Pelvic RT dose per fractionation (Gy)	Boost total dose (Gy)	Boost dose per fractionation (Gy)	Local control	Overall survival	Progression-free survival	Late toxicity (Grade 3 or worse)
Haas et al. [12]	Retrospective	6	14	50.4–61.2	1.8	19.5–20	4–6.5	100% (Overall)	100% (Overall)	100% (Overall)	0% (Overall)
Marnitz et al. [13]	Retrospective	11	6	50.4	1.8	30	6	100% (Overall)	100% (Overall)	100% (Overall)	0% (Overall)
Hsieh et al. [14]	Retrospective	9	NR	50–50.4	1.8–2	16–27	2–4.5	77.8% (3-year, LRC)	46.9% (3-year)	25.9% (3-year)	0% (Overall)
Ito et al. [15]	Prospective phase I	6	17	45	1.8	21–22.5	7–7.5	100% (Overall)	100% (Overall)	83.3% (Overall)	0% (Overall)
Albuquerque et al. [16]	Prospective phase II	15	19	45	1.8	28	7	70.1% (2-year)	53.3% (2-year)	46.7% (2-year)	26.7% (2-year)
Dalwadi et al. [17]	Retrospective	25	25	45–50.4	1.8	12–30	4–7	95.5% (2-year, LRC)	95.5% (2-year)	NR	4.0% (Overall)
Current study	Retrospective	25	34.2	44–50.4	1.8–2	20–33	4–5.5	80.9% (3-year)	77.1% (3-year)	58.5% (3-year)	20.0% (Overall)

RT, radiotherapy; NR, not reported; LRC, locoregional control

The major concern of cervical SABR boost is the potentially high toxicity due to a higher dose to organs at risk (OARs). Brachytherapy has advantages over EBRT in the dose distribution to OARs, and previous RT plan studies that tried to mimic the dose distribution of brachytherapy by IMRT and proton beam demonstrated that EBRT plans were inferior to brachtherapy plans [19]. Although several studies did not report severe late toxicities [12, 13], these studies usually had a short follow-up period with a small number of patients, making them less representative. A recent prospective phase II study reported a 2-year cumulative grade 3 or worse toxicity rate of 26.7%, which was predominantly rectal, and two of the patients had grade 5 fistula formation [16]. The study used a dose-fractionation scheme of 28 Gy with 4 fractionations, equivalent to a BED of 47.6 Gy with an α/β ratio of 10, which is relatively high. The current study had a median SABR boost BED of 37.5 Gy with an α/β ratio of 10, and although manageable, 20% of the patients experienced grade 3 toxicity. A recent prospective phase I study showed that a dose-fractionation scheme of 22.5 Gy in 3 fractions was tolerable, which is equivalent to a BED of 39.4 Gy with an α/β ratio of 10 [15]. Additional studies are needed to determine a safe and effective dose-fractionation scheme.

In the univariate analysis and ROC curve analysis of the current study, we demonstrated that cumulative D_2cc_ EQD2 of rectum was marginally correlated with the occurrence of hematochezia as a late toxicity. It can be easily assumed that the anterior rectal wall would be irradiated with high dose in both pelvic RT and SABR boost. Thus, it can be concluded from the findings of the present study that limiting the dose to the anterior rectal wall might be helpful in reducing the rectal toxicity of cervical SABR boost. The dosimetric analysis of the previously mentioned prospective phase II study showed that the percentage of the rectal circumference receiving 15 Gy was associated with the development of rectovaginal fistula [16]. In case of brachytherapy, guidelines from the American Brachytherapy Society suggest a rectal dose limit of D_2cc_ 75 Gy EQD2 for brachytherapy with 3D imaging [20]. The exact thresholds vary, but limiting the dose to some portion of the anterior rectal wall is a common feature. In contrast, for the occurrence of hematuria, cumulative D_2cc_ EQD2 of bladder showed no statistical significance in the present study. A prospective study with planned dosimetric analysis and precise assessment of adverse events is needed to determine useful OAR dose thresholds.

The present study has several limitations. Considering the small number of enrolled patients, the cohort of this study may not be representative enough to address the feasibility of cervical SABR boost. Additionally, the patients were older and more morbid compared to previous landmark studies, and this may have affected the assessment of the local control rate. Toxicity may also have been underreported due to the retrospective nature of this study. However, the present study has a relatively larger patient cohort and longer follow-up period than previous studies. Considering that cervical SABR boost is utilized secondary to brachytherapy, recruiting a large number of patients may be very challenging; consequently, the results can be considered meaningful even with this size of the cohort. Furthermore, as there is a higher chance of brachytherapy being considered not feasible in old and morbid patients, this study shows possible outcomes of patient groups in the real world.

## Conclusion

SABR boost for patients with locally advanced cervical cancer who are unable to undergo brachytherapy was found to be effective with manageable toxicity. Although it cannot substitute brachytherapy, SABR boost can be a treatment option when brachytherapy is not possible. The exact dose-fractionation scheme and dose limitation for OARs need to be addressed in further studies to ensure the safety of cervical SABR boost.

## Data Availability

The datasets used and/or analysed during the current study are available from the corresponding author on reasonable request.
